# Polymorphisms of Glutathione S-transferases Omega-1 among ethnic populations in China

**DOI:** 10.1186/1471-2156-9-29

**Published:** 2008-04-10

**Authors:** Songbo Fu, Jie Wu, Feng Chen, Dianjun Sun, Songbin Fu

**Affiliations:** 1 The Center for Endemic Disease Control, Chinese Center for Disease Control and Prevention, Harbin Medical University, Harbin, 150081, China; 2 Laboratory of Medical Genetics, Harbin Medical University, Harbin, 150081, China; 3 Bio-pharmaceutical Key Laboratory of Heilongjiang Province, Harbin 150081, China

## Abstract

**Background:**

Glutathione S-transferases (GSTs) is a genetic factor for many diseases and exhibits great diversities among various populations. We assessed association of the genotypes of Glutathione S-transferases Omega-1 (GSTO1) A140D with ethnicity in China.

**Results:**

Peripheral blood samples were obtained from 1314 individuals from 14 ethnic groups. Polymorphisms of GSTO1 A140D were measured using polymerase chain reaction-restriction fragment length polymorphism (PCR-RFLP). Logistic regression was employed to adjustment for regional factor. The frequency of GSTO1 140A allele was 15.49% in the total 14 ethnic populations. Compared to Han ethnic group, two ethnic populations were more likely to have AA or CA genotype [odds ratio (OR): 1.77, 95% confidence interval (95% CI): 1.05–2.98 for Uygur and OR: 1.78, 95% CI: 1.18–2.69 for Hui]. However, there were no statistically significant differences across 14 ethnic groups when region factor was adjusted. In Han ethnicity, region was significantly associated with AA or CA genotype. Han individuals who resided in North-west of China were more likely to have these genotypes than those in South of China (OR: 1.63, 95% CI: 1.21–2.20).

**Conclusion:**

The prevalence of the GSTO1 140A varied significantly among different regional populations in China, which showed that geography played a more important role in the population differentiation for this allele than the ethnicity/race.

## Background

Glutathione S-transferases (GSTs) constitute multifunctional enzymes that metabolize the biotransformation and disposition of a wide range of exogenous and endogenous compounds via glutathione (GSH) conjugation [1]. Seven sub-classes of GSTs have been identified in humans, including Alpha, Mu, Pi, Theta, Zeta, Sigma and Omega [2]. The Omega GSTs (GSTO), a new subfamily of the human GSTs was identified through analysis of the expressed sequence tag (EST) database and sequence alignments [2,3]. They have a cysteine residue in the active site compared to a tyrosine or serine residue in other mammalian GSTs [4]. A type of the Omega GSTs, GSTO1, has a special activity of reducing monomethylarsonic acid (MMAV) to monomethylarsonous acid (MMAIII), which appears to be the rate-limiting step in the biotransformation of inorganic arsenic [5]. As a notorious environmental carcinogen, inorganic arsenic is responsible for endemic arsenicosis because of arsenic contamination of drinking water in many regions of the world. Chronic exposures can lead to hyperkeratosis and loss of skin pigmentation as well as cancers of the skin, bladder, and lung.

Variability in human arsenic metabolism could be an underlying determinant of individual susceptibility to arsenic-induced disease in humans. A140D and T217N of GSTO1 variants reduce enzyme activity and the inhibited inorganic arsenic biotransforming capacity [6]. Some literatures reported that GSTO1 A140D substitution had a relationship with cancer, vascular dementia and stroke [7,8]. In several previous researches, the frequency of GSTO1 A allele at position 140 is different in some regions and races, such as Brazilian (f = 0.156) [9], Australian (f = 0.335), Japanese (f = 0.118), Chinese (f = 0.165) [10], German (f = 0.32) [8], African (f = 0.081), EA (European ancestry American) (f = 0.30–0.35), AA (African ancestry American) (f = 0) [11]. In this study, we analyzed distribution of the GSTO1 A140D polymorphisms among 14 ethnic populations in China.

## Results

Because the change of the GSTO1 allele C to allele A at position 140 removes one of the two *Cac8I *restriction sites in the amplified fragment, the GSTO1 140 A/A yielded 265 bp, 145 bp and 61 bp fragments, the GSTO1 140A/D yielded 410 bp, 61 bp, 265 bp and 145 bp fragments, whereas the GSTO1 140 D/D yielded 410 bp and 61 bp fragments.

After the genotype of each individual was acquired, allele and genotype frequencies of each population were calculated. Of the 1314 individuals, 24 had AA genotype, 359 CA genotype and 931 CC genotypes. The SNP genotyped was in Hardy-Weinberg equilibrium (*P*> 0.05) in our studied populations. The frequency of the GSTO1 mutant allele A at position 140 ranged from 0.0811 to 0.2203, and the mean frequency was 15.49%. The genotypic distribution varied significantly across ethnic populations (*P*< 0.05) and regions (*P *< 0.001) (see Table 1).

**Table 1:** Distribution of the GSTO1 140 mutant allele frequency in the 14 Chinese ethnic populations

		Genotype			
			
	Sample size	CC	CA	AA	Mutant allele A (10^-4^)
Ethnicity					
Han	215	158	55	2	1372
Mongol	99	74	23	2	1363
Oroqen	66	50	16	0	1212
Ewenk	45	34	11	0	1221
Korean	157	109	42	6	1719
Daur	37	31	6	0	811
Hezhen	95	64	30	1	1684
Man	103	74	28	1	1456
Kyrgyz	42	28	14	0	1667
Uygur	57	34	21	2	2192
Hui	118	72	40	6	2203
Xibo	74	51	22	1	1622
Hazakh	91	62	27	2	1703
Bouyei	115	90	24	1	1130
Region					
North-east	602	436	156	10	1462
North-west	412	267	134	11	1893
South	300	228	69	3	1250

As seen in Table 2, compared to Han ethnic individuals, two ethnic groups were more likely to have AA or CA genotype [odds ratio (OR): 1.77, 95% confidence interval (95% CI): 1.05–2.98 for Uygur and OR: 1.78, 95% CI: 1.18–2.69 for Hui]. However, the statistically significant differences across 14 ethnic groups disappeared when region factor was adjusted.

**Table 2:** Association of ethnicity and genotype CA or AA in China

	Unadjusted Odds Ratio			Region adjusted Odds Ratio		
	
Ethnicity	Odds ratio	95%confidence interval	*P*-value	Odds ratio	95% confidence interval	*P*-value
Han	1.0	(reference group)	-	1.0	(reference group)	-
Mongol	0.99	0.61~1.62	0.9772	1.03	0.62~1.72	0.8957
Oroqen	0.87	0.48~1.55	0.6366	0.90	0.49~1.65	0.7417
Ewenk	0.88	0.44~1.73	0.7050	0.91	0.45~1.84	0.7965
Korean	1.31	0.87~1.95	0.1927	1.36	0.89~2.07	0.1506
Daur	0.56	0.23~1.34	0.1889	0.58	0.24~1.40	0.2260
Hezhen	1.27	0.80~2.04	0.3121	1.33	0.82~2.16	0.2527
Man	1.07	0.67~1.72	0.7745	1.12	0.68~1.82	0.6591
Kyrgyz	1.26	0.67~2.38	0.4801	1.00	0.41~2.43	1.0000
Uygur	1.77	1.05~2.98	0.0326	1.40	0.62~3.17	0.4117
Hui	1.78	1.18~2.69	0.0063	1.41	0.67~2.98	0.3633
Xibo	1.22	0.73~2.04	0.4558	0.97	0.43~2.17	0.9366
Hazakh	1.29	0.80~2.08	0.2911	1.03	0.47~2.24	0.9477
Bouyei	0.80	0.49~1.31	0.3779	0.84	0.50~1.39	0.4855

In the samples of Han ethnic group from three regions, region was significantly associated with AA or CA genotypes (see Table 3). Han individuals who resided in North-west of China were more likely to have these genotypes than those in South of China (OR: 1.63, 95% CI: 1.21–2.20).

**Table 3:** Association of region with genotype CA or AA among Han ethnic individuals

	Odds ratio	95% confidence interval	*P*-value
South	1	(reference group)	-
North-east	1.2	0.90~1.60	0.2215
North-west	1.63	1.21~2.20	0.0012

## Discussion

We assessed ethnic variation of the GSTO1 A140D mutant allelic frequency among 14 Chinese ethnic populations. The genotypic distribution varied across regions, which support the previous study. It has been reported that genetic structure, particularly the Y chromosome and mitochondrion study differed by regions [12,13]. As different groups belong to different regions, the variances of genetic structure between different groups lay on their large geographical span.

It is demonstrated that the observed ethnic variation was not significant once geographic region was accounted for. We further evidenced that region was associated with the genotype. These findings support that genotype of CA and AA distribution is likely to be determined by environmental factors rather than ethnicity.

Our study further demonstrated the regional difference appeared the comparison between North-west and South of China. The result suggests that environmental factors play a significant role in the formation of genetic characteristics in Chinese populations. Migration between north-west and the south of China might be rare in ancient time because of lack of transportations to overcome numerous huge mountains and other barriers. That might inhabit gene flow across regions. On the other hand, most of the northern areas are plains. This geographic character, as well as the long history of wars and famines in the north, may have contributed to plentiful gene flows among the northern populations. Populations from northwest of China showed comparatively higher mutation allele frequency, probably due to the ancestral gene flow from Europe and recent intermarriage amongst the northwest groups.

By further literature review of the allelic frequencies in the world, we found the prevalence of the allele varied substantially between continental populations. For example, the frequency of GSTO1 140A is 0.32 in German, [8] 0.30–0.35 in European ancestry American [11], 0.118 in Japanese and 0.165 in Chinese [10], but only 0.081 in African population and even absent in African ancestry American [11]. The present results of variation of the SNP frequencies with geographical regions are mainly attributable to the different population historical events of the geographically separated population groupings.

This study has several limitations. Firstly, besides A140D substitution, there are several other common polymorphisms that can induce change of amino acids in GSTO1 exons. In those polymorphic sites, E208K and E155del exist polymorphisms in the Chinese [10] and both mutant frequencies are very low. We could not obtain the accurate data on allele distribution of these two polymorphisms in our samples. Secondly, we only included 14 ethnic populations out of 56 ethnic populations. However, it is less likely to observe significant ethnic variation even if more ethnic groups were recruited. Thirdly, we also studied T217N and A236V sites in relatively large ethnic populations of Hui, Mongol, Sichuan Han and Hazakh. The additional analysis did not find any polymorphism. Therefore, it is necessary to enlarge sample sizes for other ethnic samples.

## Conclusion

The prevalence of the GSTO1 140A varied significantly among different regional populations in China, supporting that geography played a more important role in the population differentiation than ethnicity or race. To some degree, our study findings suggest that ethnicity not be considered in the study of causal relationship between genetic polymorphisms and disease.

## Methods

### Samples

**Figure 1:**
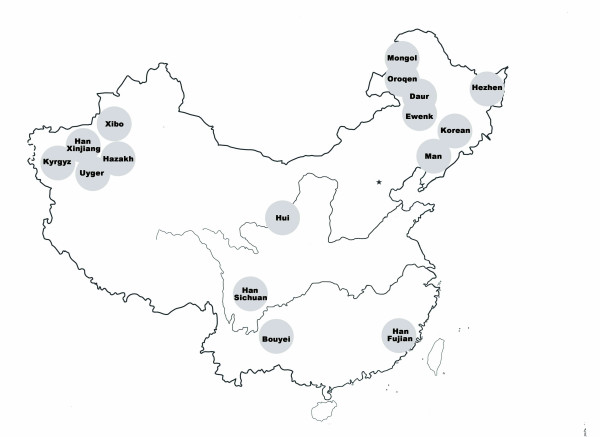
Geographic distribution of study populations in China.

There are 56 ethnic populations in China. Han is the largest ethnic group, accounting for 95% population in China. A majority of remaining 55 ethnic population are residing in remote areas and some reside in ethnic communities. We divided the geography of China into southern and northern regions and then further divided northern China into north-east and north-west. We identified ethnic communities in these three regions (i.e. south, north-east and north-west of China) and randomly selected communities/villages. For the 16 groups involved in the present study, Fujian Han, Sichuan Han and Bouyei were sampled in south of China. Mongol, Man, Korean, Ewenk, Oroqen, Daur, and Hezhen were sampled in north-east of China and Uyger, Kyrgyz Hazakh, Hui, Xibo and Xinjiang Han were sampled in north-west of China. All ethnic residents in the selected communities were screened through face-to-face survey for identifying individuals who were healthy and from a family without inter-racial/ethnic marriage in the last three generations. We invited those who met these criteria to participate in the study. For families with more than one person including siblings, parents and relatives, we randomly selected only one person to ensure no genetic linkages among recruited individuals. Peripheral blood samples were collected from those who signed the informed consent after explanation of study purposes and process. The study protocol was ethically approved by the Ethics Committee of Harbin Medical University, China. The final sample size was 1314 individuals, Figure 1 and Table 4 showed detail information of the 14 involved ethnic population distribution. We selected Han ethnic samples from three different regions to test regional variation in one ethnic population. We expected to recruit at least 50 persons for each population.

**Table 4:** Geographic location of study population

Population	Sample size	Sampling location	Longitude and latitude
	90	Minqing County, Fujian Province,	118.3, 25.6
Han	95	Chengdu City, Sichuan Province,	104.1, 30.6
	30	Yining, Xinjiang Autonomous Region	80.5, 43.3
Oroqen	66	Arlihe, Inner Mongolia Autonomous Region	131.0, 53.5
Ewenk	45	Molidawa, Inner Mongolia Autonomous Region	118.6, 48.0
Korean	157	Yanji City, Jilin Province	121.4, 40.2
Daur	37	Molidawa, Inner Mongolia Autonomous Region	118.6, 40.8
Hezhen	95	Tongjiang, Heilongjiang Province	132.5, 46.7
Man	103	Xiuyan City, Liaoning Province	123.2, 40.2
Kyrgyz	42	Wuqia, Xinjiang Autonomous Region	73.4, 39.2
Uygur	57	Yining City, Xinjiang Autonomous Region	80.5, 43.3
Mongol	99	Hailar City, Inner Mongolia Autonomous Region	126.0, 51.0
Hui	118	Tongxin County, Ningxia Autonomous Region	105.9, 36.9
Xibo	74	Yili, Xinjiang Autonomous Region	80.5, 43.3
Hazakh	91	Yining City, Xinjiang Autonomous Region	80.5, 43.3
Bouyei	115	Guizhou City, Yunnan Province	105.7, 26.0

### Genotyping assay

Genomic DNA was extracted from peripheral blood anti-coagulated with ACD by a standard phenol-chloroform protocol. GSTO1 140 variants were studied by polymerase chain reaction-restriction fragment length polymorphism (PCR-RFLP). PCR was performed in 20 ul volumes of a mixture containing 50 ng DNA template, 0.2 uM each primer, 200 uM dNTPs, 1.5 mM MgCl_2_, 50 mM KCl, 10 mM Tris-HCl (pH 8.3) and 2.0 U Taq DNA polymerase (Takara). The forward and reverse primers are 5'-aacgtgggtacaatttcc-3' and 5'-ccaggactgtaagggttc-3', respectively. Amplification was carried out for 32 cycles at 94°C 30 s, 55°C 30 s and 72°C 30 s with Eppendorf AG. Then the PCR products were digested with *Cac8I *restriction endonuclease (5'-GCN/NGC-3') at 37°C for 14 h, and the digested products were detected on 2.5% agarose gels.

### Statistical analysis

Deviations from Hardy-Weinberg equilibrium (HWE) were investigated for this locus using the chi-square statistic, with expected frequencies derived from allele frequencies. We calculated the allele and genotype frequencies by direct counting and tested the significant differences among the populations using the chi-square. Logistic regression was fitted using genotype 'AA or CA' as dependent variable, and ethnicity and regions as independent variables to test variation of genotype across ethnicity after adjustment for regions. Data were analyzed using SAS software (version 8.2; SAS Institute Inc., Cary, NC, USA).

## Authors' contributions

Songbo Fu led study design and drafted and revised this manuscript. Jie Wu recruited samples, obtained blood samples and performed the PCR-RFLP experiments. Feng Chen contributed to statistical analysis. Songbin Fu and Dianjun Sun initiated the study, and participated in its design and coordination and involved in the drafting the manuscript. Besides the specific contributions to the paper, all authors contributed to data interpretation, revising manuscripts and and approving the final manuscript.
